# Supplementary material for: Assessing key decisions for transcriptomic data integration in biochemical networks
Source: PLoS Comput Biol. 2019 Jul 19;15(7):e1007185. doi: 10.1371/journal.pcbi.1007185 (PMC6668847; doi:10.1371/journal.pcbi.1007185)
Supplement: S1 Text — (DOCX) [file pcbi.1007185.s001.docx]

**S1 Text -Assessing key decisions for transcriptomic data integration in biochemical networks**

*Anne Richelle, Chintan Joshi, Nathan E. Lewis*

**
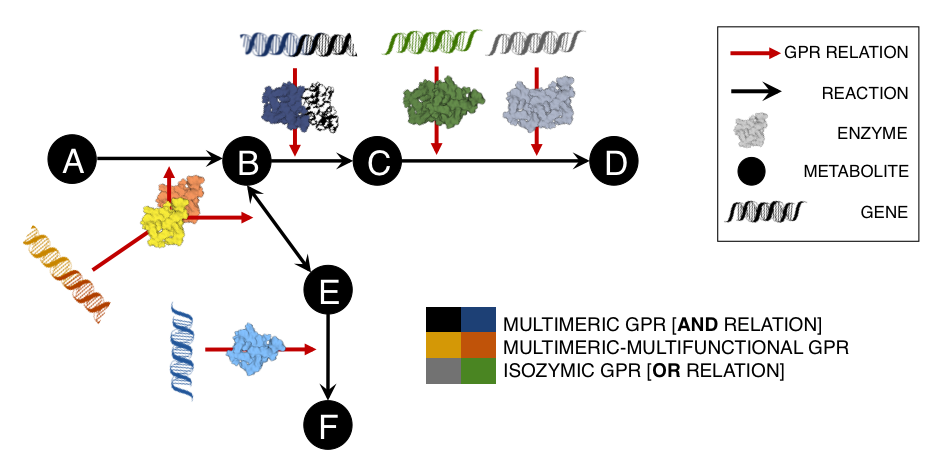
**

**Fig. A** - GPR relationships describe how genes and enzymes link to reactions: (1) one enzyme can catalyze one reaction (light blue), (2) multiple proteins subunits forming an enzyme catalyze one reaction (multimeric GPR, dark blue and black), (3) one enzyme formed by multiple proteins subunits catalyze multiple reactions (multimeric – multifunctional GPR, yellow and orange), (4) multiple enzymes could catalyze one reactions (isoenzymic GPR)


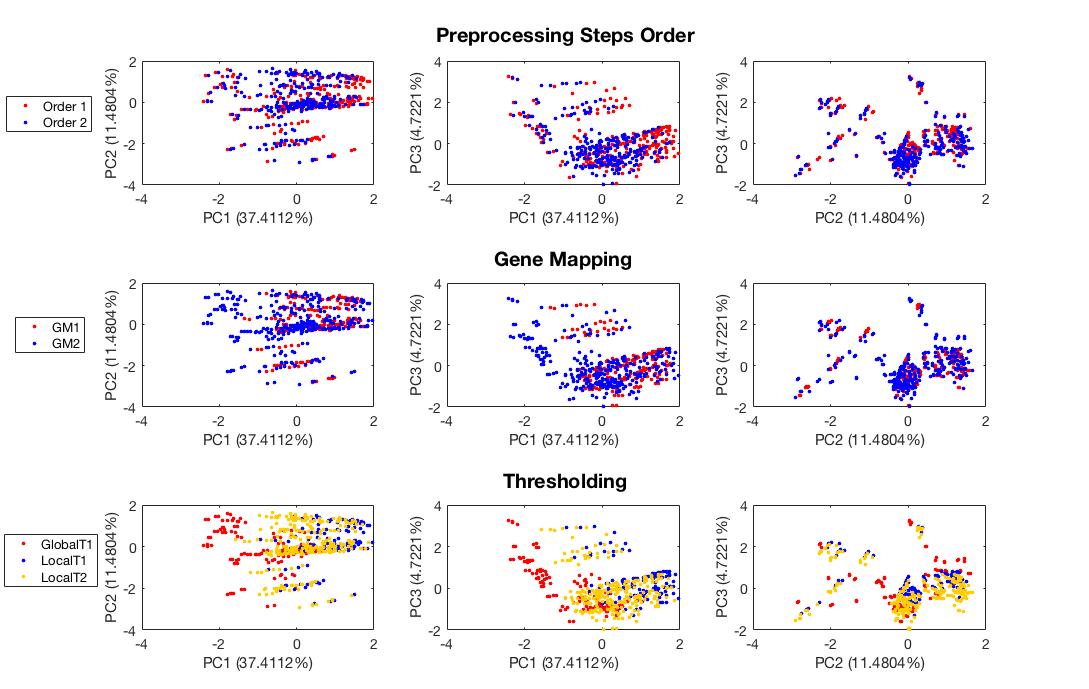


**Fig. B** - PCA of reaction set considered as active. Principal component scores of the first principal component (PC1) versus the scores of the second (PC2) and the third principal component (PC3). Axis labels indicate which component scores are plotted. Figure titles indicate the preprocessing decisions and whether data point are colored with respect to the respective variables for each preprocessing decision.


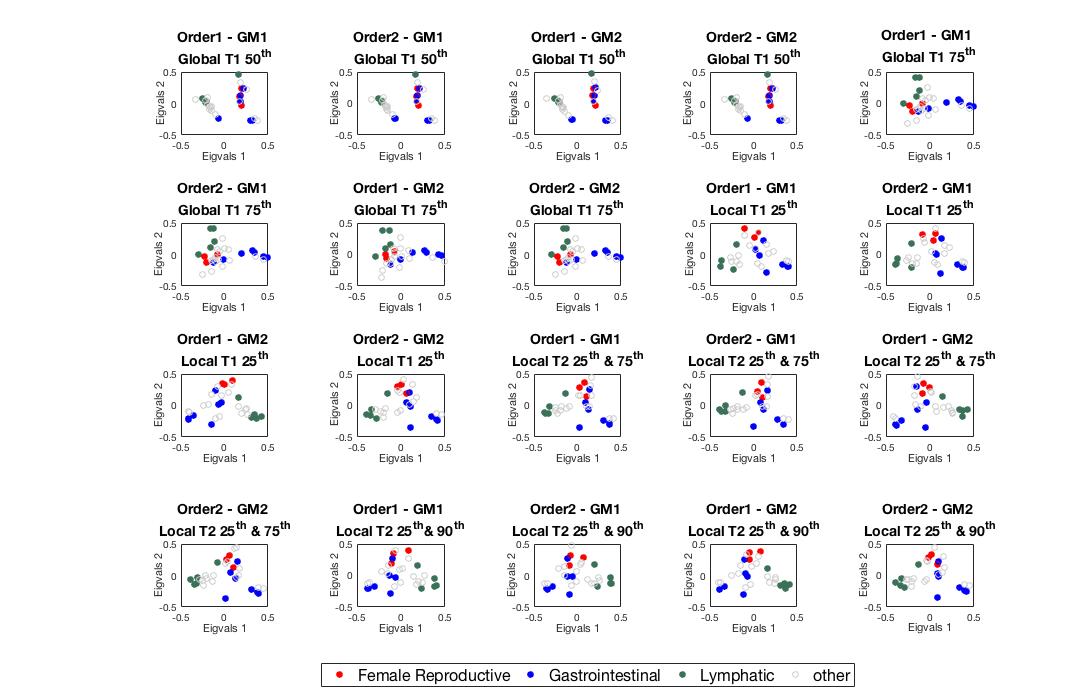


**Fig. C -** Tissue similarities for each combination of preprocessing decision visualized using a Principal Coordinates Analysis (PCoA). Title of the plots correspond to the preprocessing combination and axis to the two first principal coordinates.


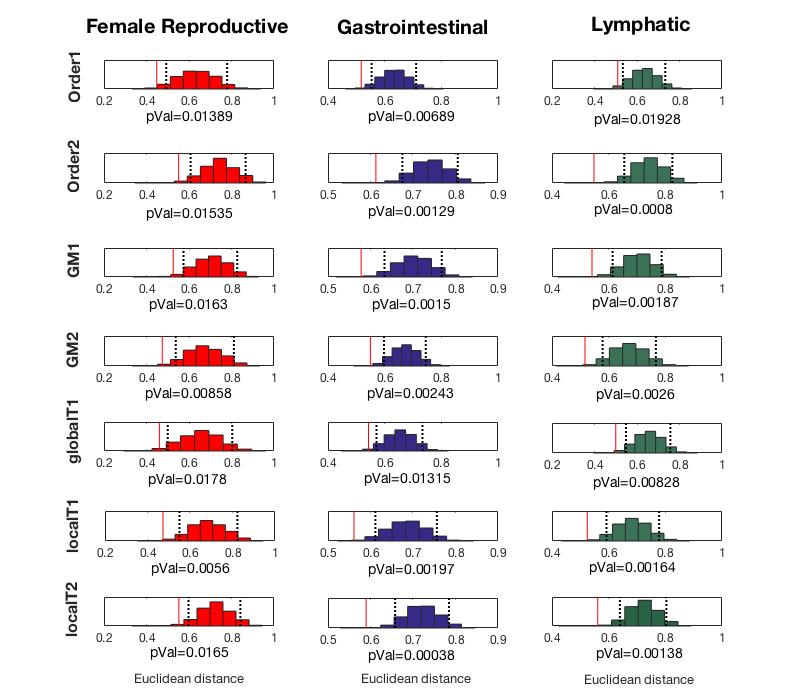


**Fig. D -** Significance of tissue grouping at the organ-system level. Distribution of mean Euclidean distance for 100000 randomly selected groups with the same number of tissues and their associated 5^th^ and 95^th^ percentiles (black dotted lines). The red dotted line is the mean Euclidean distance observed and its associated p-value.

**
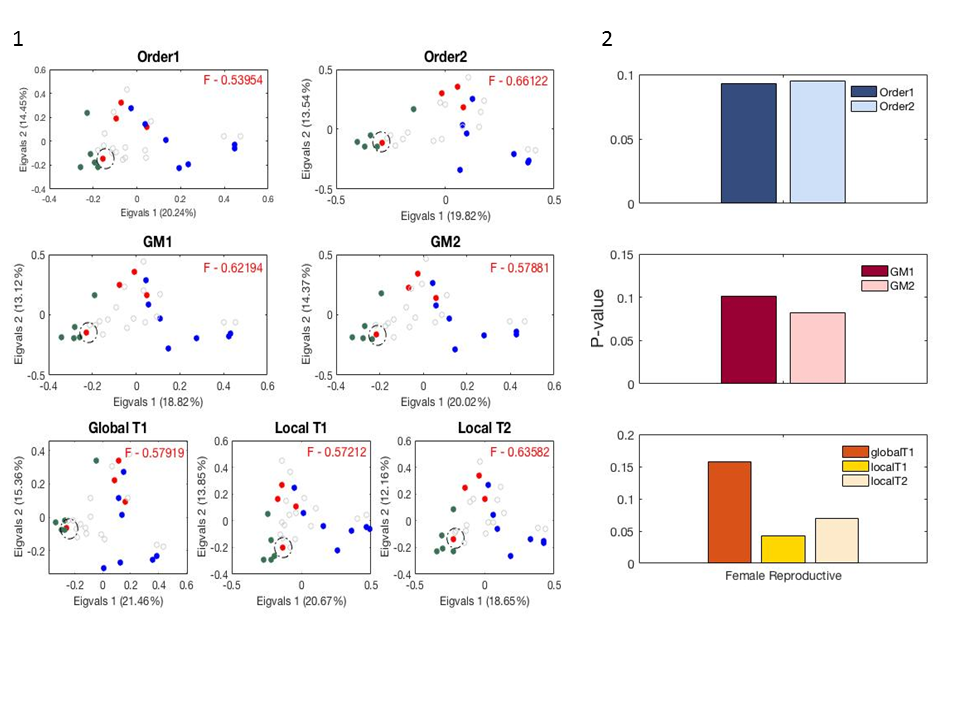
**

**Fig. E -** The grouping of tissue belonging to “*Female Reproductive*” organ-system is significantly influenced by the inclusion the placenta tissue (encircled red dot). (1) Tissue similarities for each combination of preprocessing decision visualized using a Principal Coordinates Analysis (PCoA). The Euclidean distance associated to the Female Reproductive group is increased compared the one observed without including placenta tissue (Fig. 3). (2) The grouping of “*Female Reproductive*” organ-system is no longer significant when introducing the placenta tissue (increased p-values associated to the mean Euclidean distance observed for this group compared to Fig. 4), thus highlighting the metabolic similarities among similar tissues with similar functions.


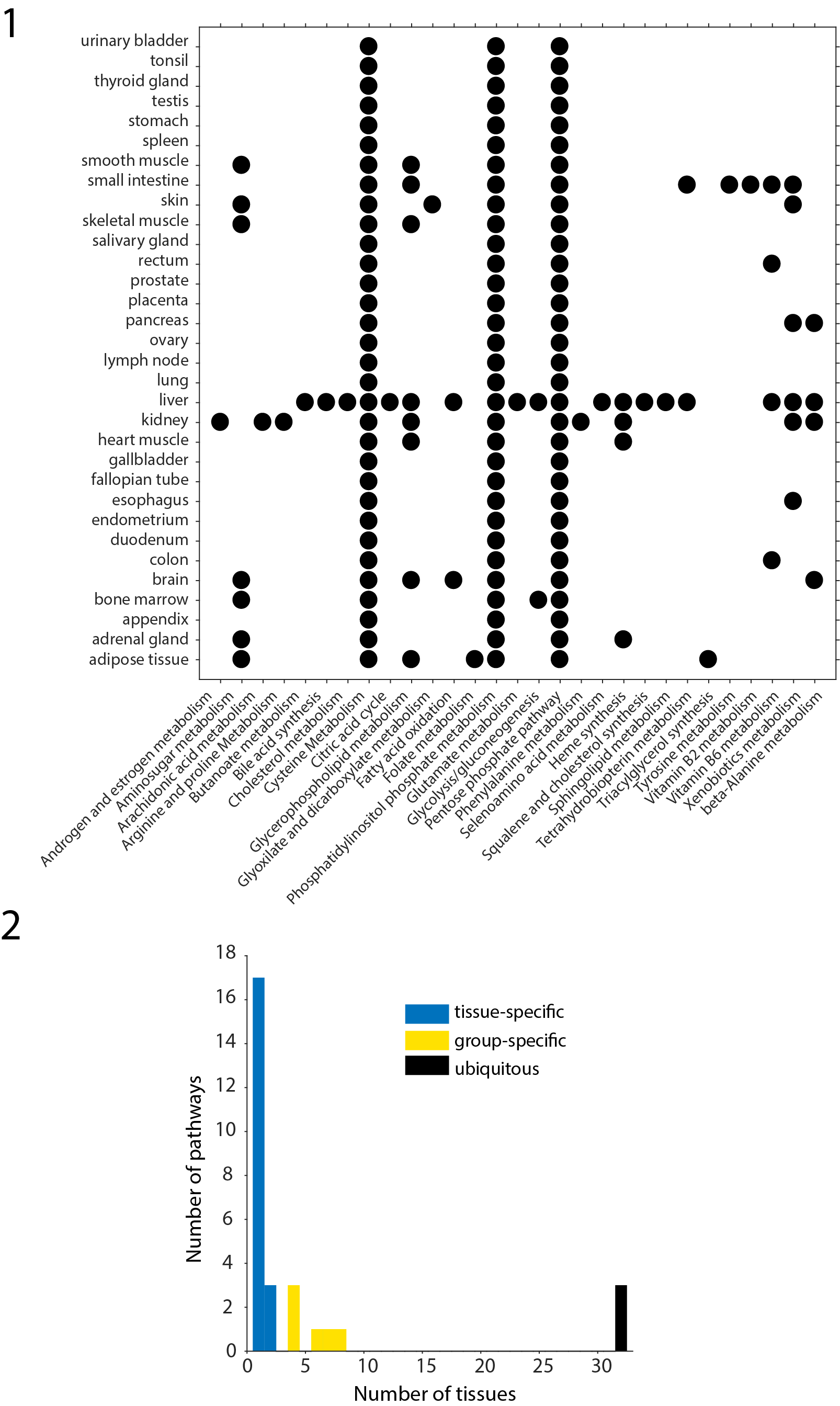


**Fig. F** - Pathways known to occur in different tissues in manually-curated resource**.** (1) Black dots indicate which pathways (x-axis) are occurring in which tissues (y-axis). (2) Pathways were divided into three categories: tissue-specific (blue, < 2 tissues); group-specific (yellow, 3 to 10 tissues); and ubiquitous (black, most or all tissues) using the manually-curated resource matrix in (1).


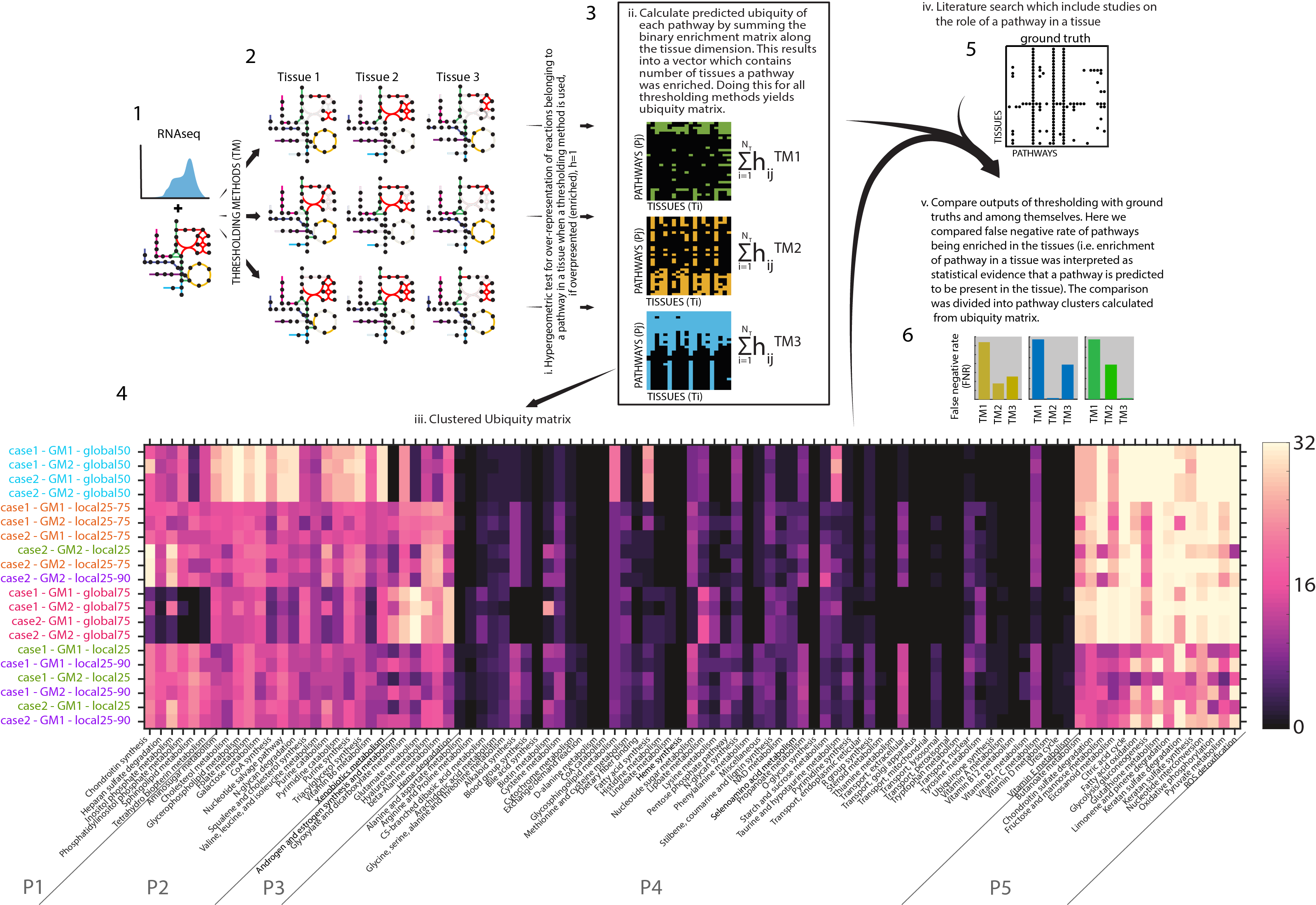


**Fig. G** - **Schematic of analyses comparing performance of thresholding methods with the manually-curated resource.** (1) RNAseq data were integrated with the metabolic network using different thresholding methods to generate a list of active reactions for each tissue (2). A toy network is represented in (1, bottom panel) where the colors indicate different pathways. (2) The reactions that were selected when a thresholding method was used is given by colors while those not selected are in gray. (3) Hypergeometric test was performed for each tissue from a given thresholding method to see if reactions selected from a pathway are over-represented. Each color represents a different thresholding method. The colored boxes in the binary heatmap represents which pathway was enriched in which tissue. (4) Doing this for all the methods and then summing it results into a matrix which describes the predicted ubiquity of pathways using each of the thresholding methods. The pathways were divided into 5 clusters (P1, P2, P3, P4, and P5) based on their predicted ubiquity. (5) Manually-curated resource of pathway-tissue pairs was created by mining from literature (Fig. F1 in S1 text). (6) The ubiquity and false negative rate are compared using the manually-curated resource for each thresholding schemes for each of the pathway clusters identified in (4). The figures of the actual analysis are shown in Figs. H and I in S1 Text.


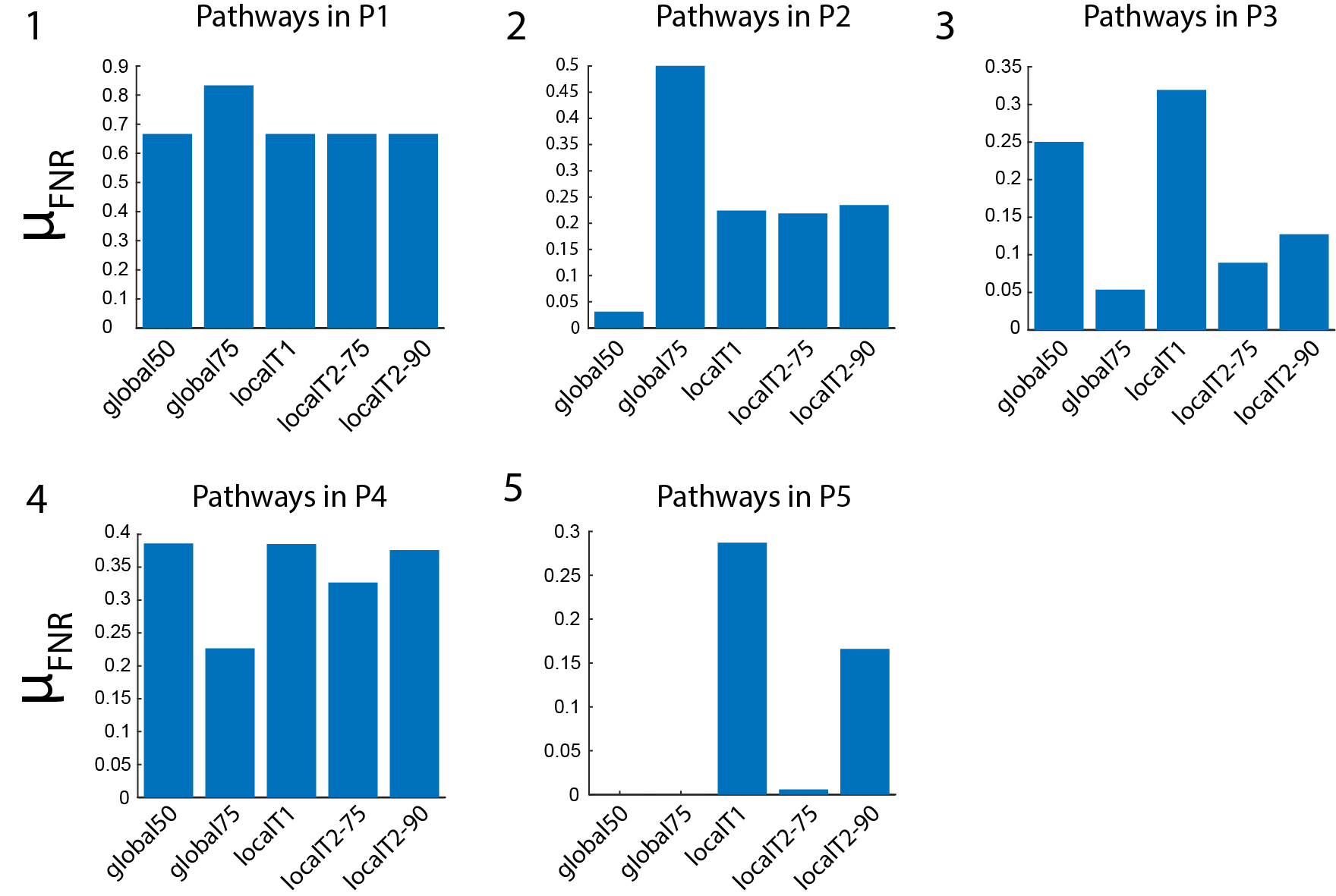


**Fig. H** - Comparison of mean false negative rate (FNR, across step-order and gene-mapping) calculated using the manually-curated resource for each of the thresholding method is shown for (1) P1, (2) P2, (3) P3, (4) P4, and (5) P5. For P2, global75 generates largest FNR predictions (2). For P3, localT1_25 (localT1) and global50 generates largest FNR predictions (3). For P5, localT1_25 (localT1) and localT2_25_90 (localT2_90) generates largest FNR predictions. Combining results for P2, P3, and P5 suggests that localT2_75 (localT2_25_75) performs best across these pathways. The Wilcoxon pair-wise test for difference in FNR distributions is shown was not significant in P1 and P4, while was significant in the other three clusters (Fig. I in S1 Text).


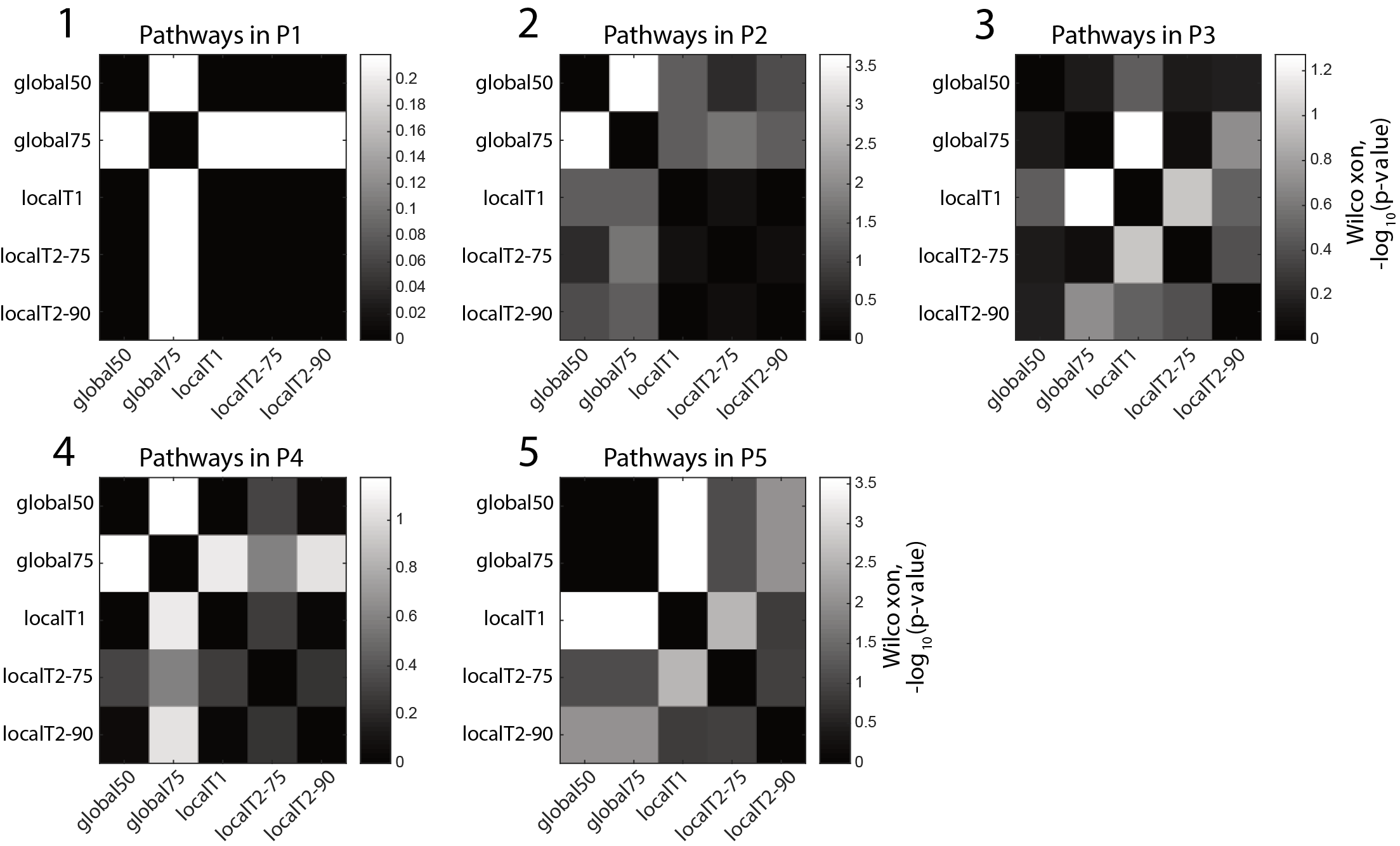


**Fig. I** - Wilcoxon pair-wise rank-sum test for testing significance in FNR distributions (Fig. H in S1 Text) between the thresholding methods for each of the pathway clusters (1) P1, (2) P2, (3) P3, (4) P4, and (5) P5. The Wilcoxon pair-wise test for difference in FNR distributions is shown was not significant in P1 and P4, while was significant in the other three clusters.


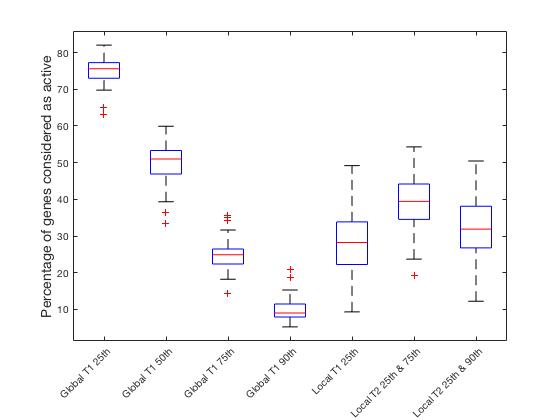


**Fig. J -** Boxplot of the percentage of genes considered as active in different tissues depending on the combinations of thresholding variables imposed

*
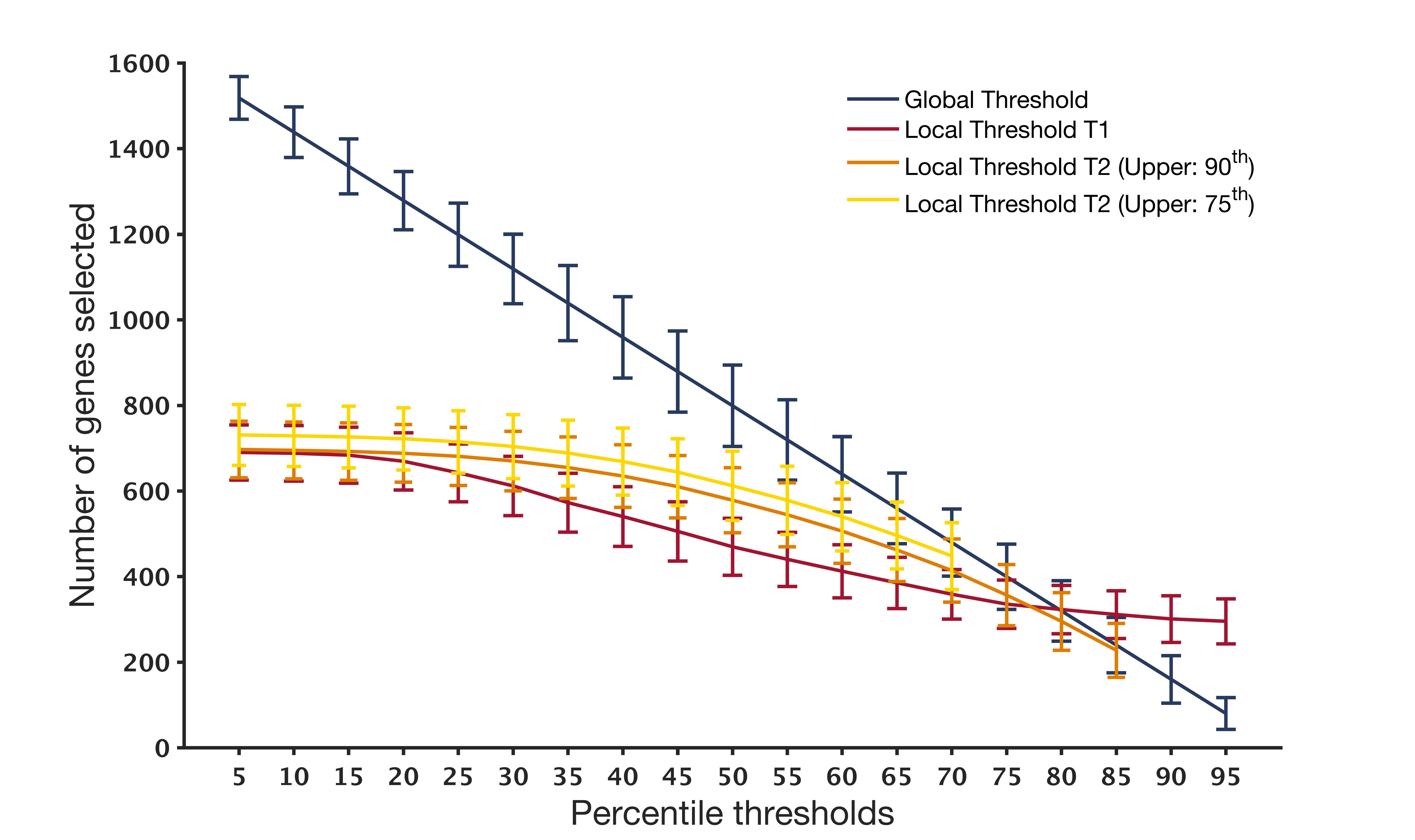
*

**Fig. K -** Mean number of genes selected when using different thresholding approach, state and value. Global approach (global T1, blue) causes large variability while local threshold (local T1, green; local T2 with upper threshold as 90 percentiles, red; local T2 with upper threshold as 75 percentiles; yellow) causes less variability. For T1, threshold is given by the value in x-axis. For T2, lower threshold is given by the value in x-axis.


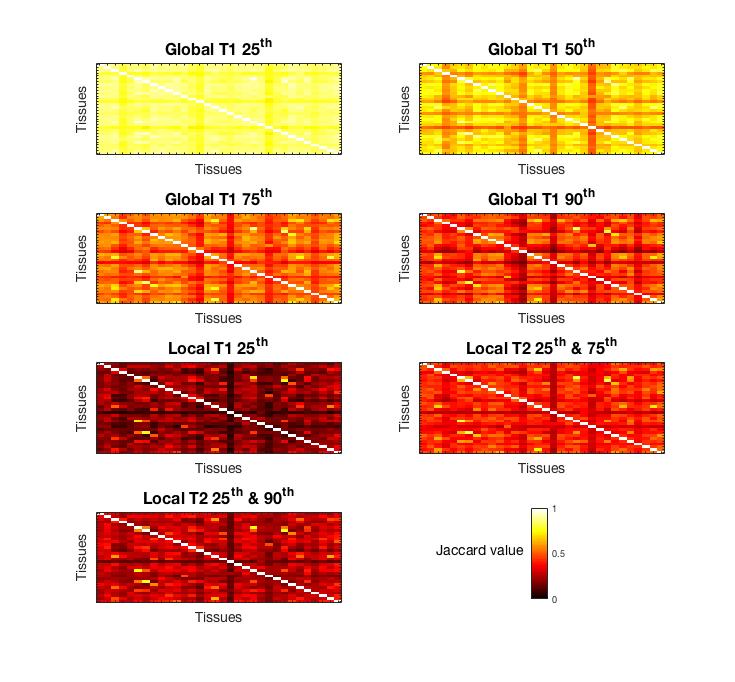


**Fig. L -** Jaccard distance similarity across tissues for different combinations of thresholding variables


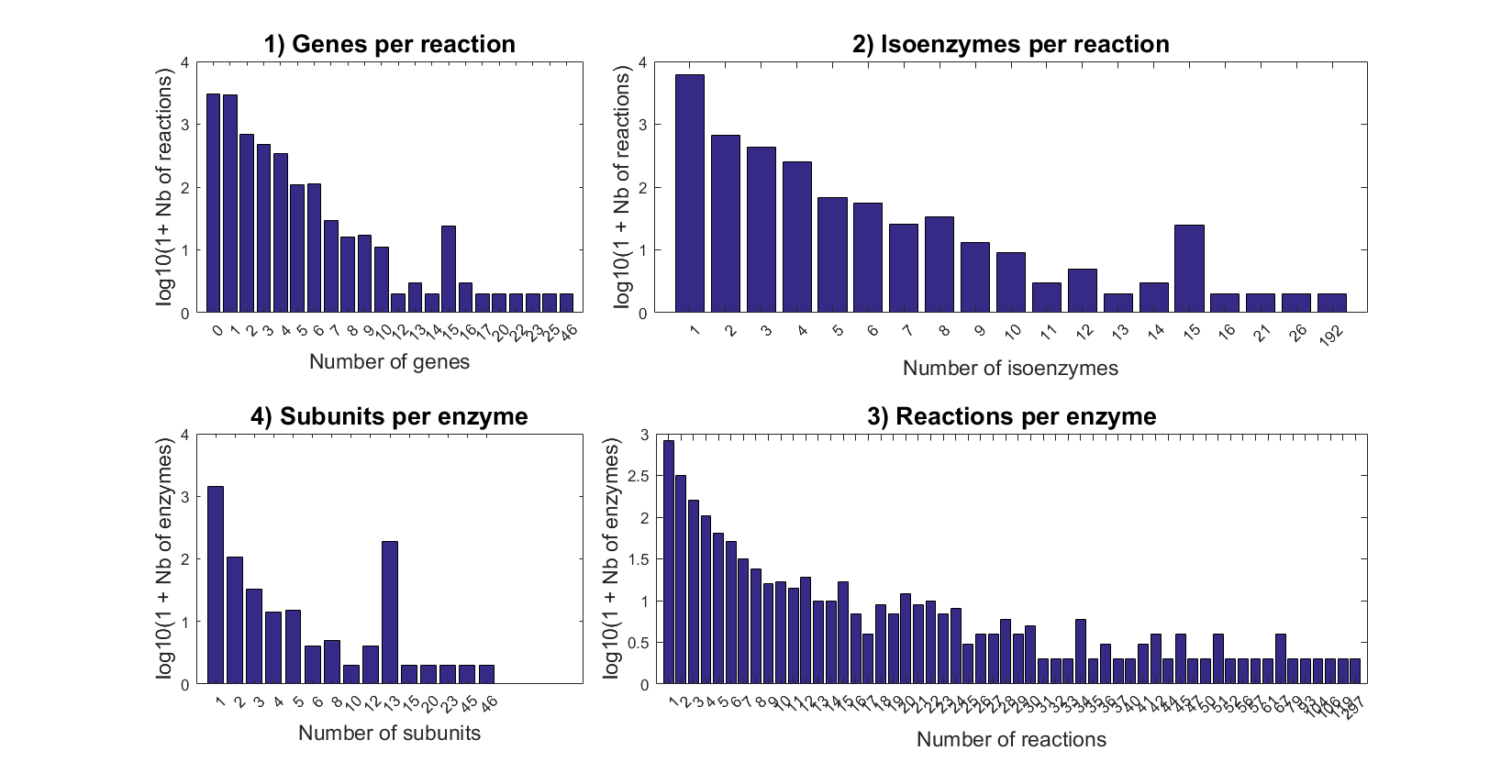


**Fig. M -** Complexity of GPR associations. Frequency distribution of the GPR associations in Recon 2.2 model with respect to: (1) number of genes per reaction; (2) number of isoenzymes per reactions; (3) number of subunits per enzyme complex; (4) number of reactions catalyzed by enzyme

**
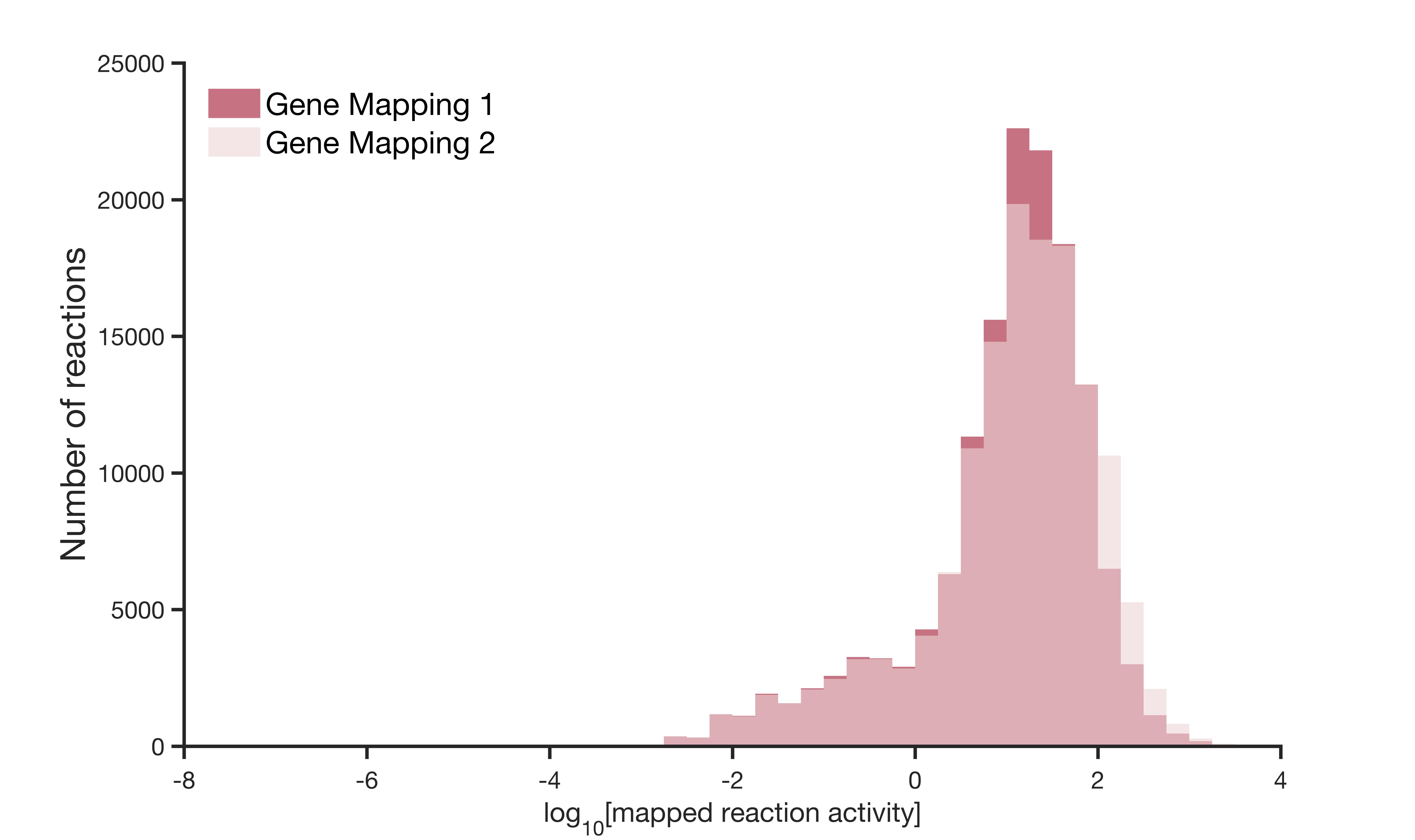
**

**Fig. N -** Distribution of reaction activity changes depending upon the choice of gene mapping method. GM2 (red) presents an increased number of reactions in the right tail of the distribution than GM1 (blue) but the distributions are generally similar.


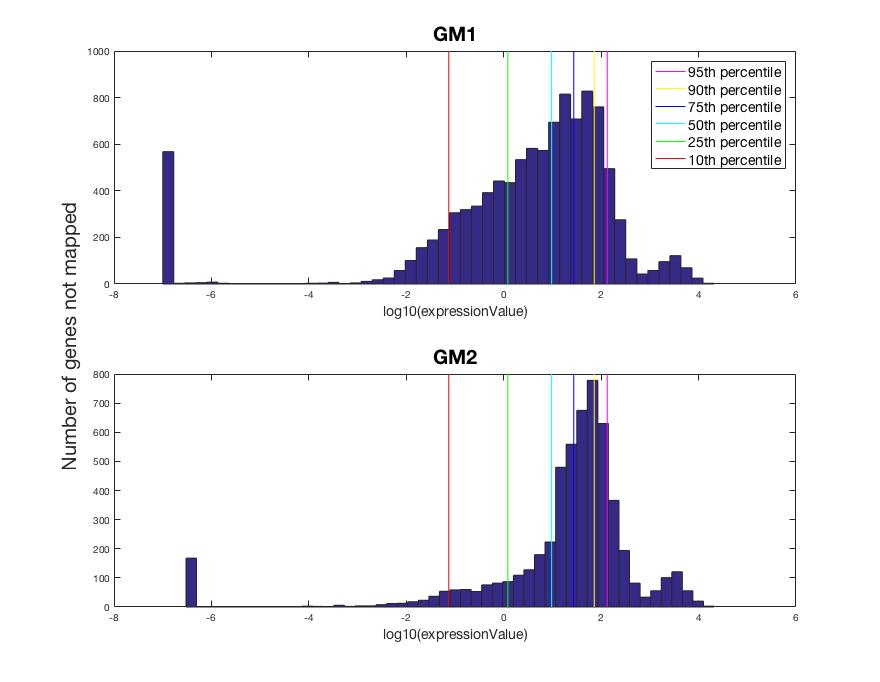


**Fig. O** - Histogram of gene expression values of genes not mapped using GM1 (top) and GM2 (bottom). Percentiles have been defined based on the distribution of all metabolic genes present in HPA dataset (same percentiles as Fig. L in S1 Text)


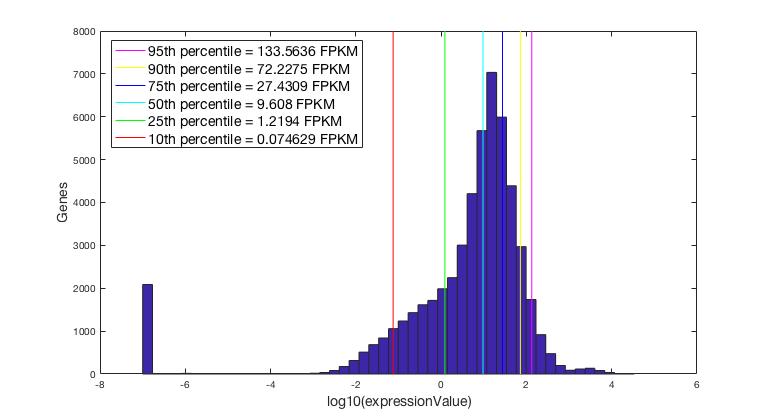


**Fig. P -** Histogram of gene expression values of all metabolic genes (1663) present in HPA dataset


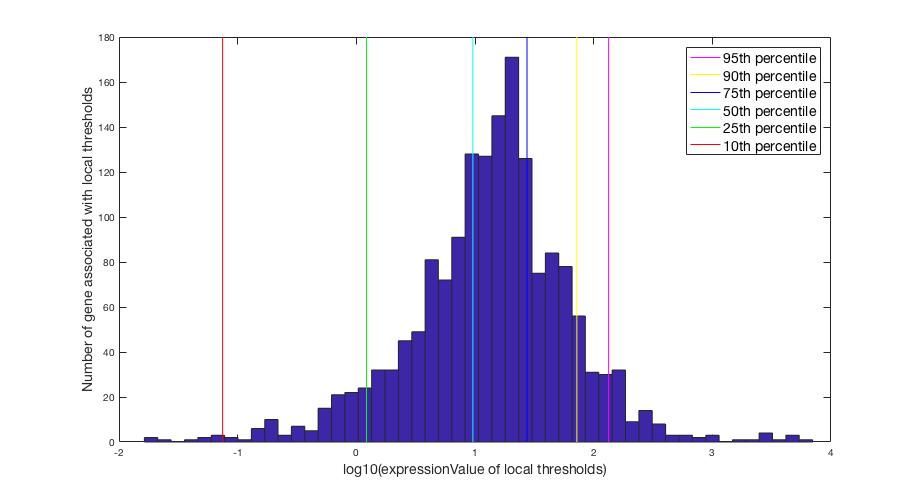


**Fig. Q -** Comparison of distribution of the mean gene expression value for each gene (local threshold) to the global threshold values defined thanks to the percentiles of overall metabolic gene expression distribution (vertical lines, same percentiles as Fig. L in S1 Text)
